# Effects of protein intake on glucagon, insulin, and glucose dynamics: implications for diabetes

**DOI:** 10.3389/fcdhc.2025.1712506

**Published:** 2026-01-12

**Authors:** Sarah Nagy, Lauren V. Turner, Michael C. Riddell

**Affiliations:** School of Kinesiology and Health Science, York University, Toronto, ON, Canada

**Keywords:** carbohydrate, diabetes, exercise, glycemia, hypoglycemia, insulin resistance, protein

## Abstract

Carbohydrates are the main macronutrient of interest for dosing insulin and managing glycemia in type 1 diabetes (T1D) due to their direct impact on blood glucose levels, however, the influence of protein on glycemia and pancreatic islet hormone secretions in people living with either T1D or type 2 diabetes (T2D) should not be overlooked. Protein ingestion plays a key role in the secretion of both insulin and glucagon, making it a key regulator of blood glucose levels in health and diabetes. The glycemic response to protein is affected by many factors including the protein’s form, source, digestion rate, whether it is consumed on its own or in a mixed meal, as well as its timing in relation to other meals and/or physical activity. Additionally, the hormonal and glycemic response to protein differs markedly between non diabetic individuals, T1D, and T2D. The unique ability of protein to modify post-prandial glycemia makes it a potential tool that individuals with diabetes or prediabetes can utilize to help manage their own glycemia. This review will discuss the ways in which protein intake and supplementation with certain protein types may be able to improve overall glycemia and time in range for individuals living with diabetes or prediabetes.

## Graphical Abstract

**Figure:**
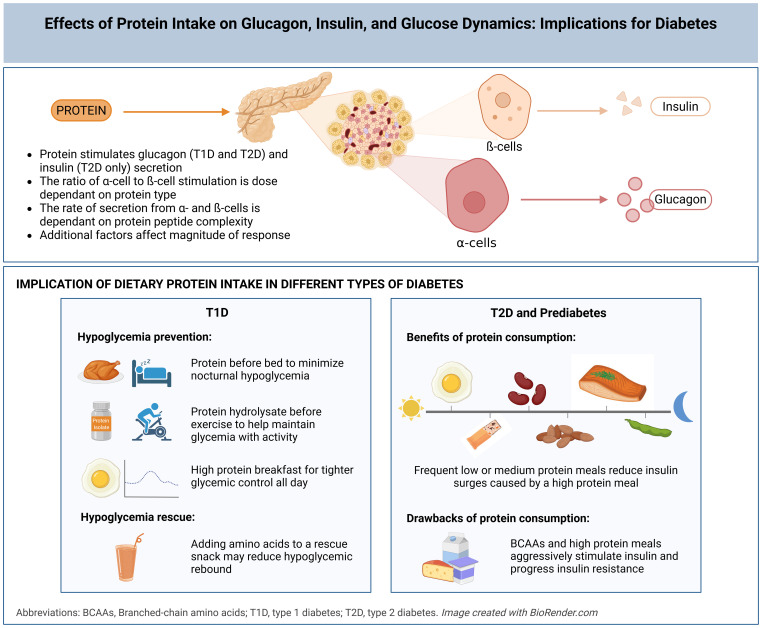

## 1 Introduction

Diabetes mellitus is a growing global health concern with increasing prevalence (1, 2). Both type 1 diabetes (T1D) and type 2 diabetes (T2D) involve dysregulated blood glucose control, but these two related diseases arise from distinct mechanisms, and have very different hormonal profiles at the time of diagnosis. In T1D, the autoimmune destruction of one’s pancreatic beta cells inhibits completely (or near completely) their ability to produce and secrete endogenous insulin, causing a lifelong reliance on exogenous insulin therapy for survival after diagnosis. In T2D, reduced insulin sensitivity and a relative deficiency in insulin secretion, and sometimes hyperglucagonemia, are more characteristic of the early disease phenotype. Treatment for individuals with T2D includes lifestyle changes, various oral medications that lower blood glucose levels, and/or exogenous insulin therapy to regain management of their fed and fasted glycemia.

Despite differences in the pathophysiology, individuals with either type of diabetes are advised to achieve >70% time in target glycemic range (TIR: 70–180 mg/dL or 3.9-10.0 mmol/L) to help prevent diabetes-related complications (3). More recently, it has been proposed that these populations may benefit from further limiting glucose fluctuations by increasing the proportion of time spent in a so called ‘normal’ or tight glycemic range (TITR: 70–140 mg/dL or 3.9-7.8 mmol/L) (4). While achieving this reduced variability may be challenging, doing so could further minimize diabetes-related complications for those with T1D (5) and T2D (6, 7); therefore, lifestyle and nutritional strategies should be considered in combination with medications to support improved glycemic management in these populations.

Nutritional recommendations in diabetes currently focus on reducing simple carbohydrates and increasing fiber intake to help manage body weight, lipid levels, and glycemia in T1D and T2D (8). However, blood glucose levels are influenced by a number of factors other than carbohydrate intake, and it is now accepted that all dietary macromolecules are important to consider due to their unique effects on glycemia (9–11). Dietary protein in particular has become a focal point in diabetes research and clinical care (and on social media), due to its unique influence on the pancreas and apparent ability to help improve overall glycemic management (12). It is important to emphasize that protein ingestion normally plays a key role in the secretion of both insulin and glucagon, making it a key regulator of blood glucose levels (13–15). Trying to emulate this normal physiology in diabetes remains an ongoing challenge-, however, because of the complexity of the various metabolic effects of certain protein types.

Protein ingestion normally stimulates alpha and beta cells to produce glucagon and insulin, respectively in people not living with diabetes (16, 17). As these hormones have opposing effects on blood glucose, the net glycemic effect of protein intake may depend on the ratio of circulating glucagon and insulin after consumption. However, the rates of insulin and glucagon secretion in response to protein depends on many factors including, but not limited to: the protein’s form, source, and digestion rate, whether it is consumed on its own or in a mixed meal, its timing in relation to other meals and/or physical activity, and the presence or absence of diabetes (18–27). As such, the interplay of these and other considerations likely determine the time profile dependent glycemic response to protein. Protein’s unique ability to differentially modify post prandial glycemic responses makes it a potential tool that could help individuals with diabetes (or prediabetes) manage their blood glucose levels after meals or when they perform physical activity, with the former situation requiring more insulin secretion to limit hyperglycemia, and the later typically requiring less insulin secretion and a significant rise in glucagon secretion into the hepatic portal vein to facilitate hepatic glucose production to help limit exercise-induced hypoglycemia. When used in different ways for people living with diabetes, protein may help blunt postprandial glucose spikes (28), extend euglycemia (29), and/or prevent hypoglycemia in the late post meal state (22, 30, 31).

The endocrine response to protein consumption is altered in diabetes. When individuals with untreated T2D underwent an arginine infusion test, they displayed higher glucagon and lower insulin secretion compared to nondiabetic controls, leading to substantially higher blood glucose levels (21). When the same individuals with T2D repeated the arginine infusion test after eight weeks of diet, insulin, or sulfonylurea therapy, their hormonal responses were improved from their own pre-treatment levels (i.e., lower glucagon release and higher insulin release, resulting in lower blood glucose), but remained worse than the non diabetic controls. While all three treatment types increased the insulin: glucagon ratio in those with T2D by attenuating the exaggerated glucagon response to arginine, and by improving the blunted insulin response, individuals with T2D still displayed a reduced insulin: glucagon ratio compared to nondiabetic controls (21). Within the same study, individuals with untreated T1D displayed significantly reduced blood glucagon levels and reduced blood glucose excursions after eight-weeks of exogenous insulin therapy when subjected to the same arginine infusion tests (21). While preliminary, and not easily feasible because of the need to perform arginine infusions, these findings suggest that the glycemic and endocrine responses to protein intake is likely different between individuals with and without diabetes, and that the impaired response in diabetes may be partially, but not fully, attenuated by diet, insulin, or sulfonylurea therapy.

Individuals with T1D, T2D, or prediabetes all have unique endocrine profiles and thus, require different strategies to maintain euglycemia. In T1D, the absence of endogenous insulin secretion means that protein ingestion can be an effective way to boost glucagon levels, without a corresponding rise in insulin. Because individuals with T1D rely on exogenous insulin, protein ingestion can therefore have an unopposed glucagon effect as long as a recent insulin bolus injection (or infusion) has not occurred. This response can be beneficial in situations of higher hypoglycemia risk- such as during prolonged exercise or during the overnight period when insulin requirements fall (32). It has long been known that the glucagon response to protein intake is preserved in T1D, even when the counterregulatory processes of glucagon to hypoglycemia or exercise are diminished (31, 33). Thus, protein can be used in various ways to reduce the risk of nocturnal hypoglycemia (34), as well as hypoglycemia induced by physical activity (22, 30), periods of fasting (29), and potentially even from excessive exogenous insulin administration (31). However, this same unopposed glucagon effect can also contribute to delayed postprandial hyperglycemia, particularly after larger protein loads or mixed meals, due to increased hepatic gluconeogenesis occurring in the absence of dynamic insulin secretion/delivery.

On the other hand, endogenous insulin secretion may be intact in individuals with T2D, and nutritional guidelines for this population aim to minimize postprandial insulin surges, as a high blood insulin concentration may promote insulin resistance and/or excessive weight gain (35). As such, protein’s stimulatory action on endogenous insulin secretion should not be overlooked. Thus, in settings of high protein intake in T2D, both insulin and glucagon secretion may rise, but the magnitude of rise of each hormone depends on the type of protein consumed.

The remainder of this review will highlight ways in which individuals with both T1D and T2D may be able to utilize protein in a variety of unique ways to help manage their glycemia.

## 2 Effects of protein form on post-meal glycemic responses

**Figure 1:**
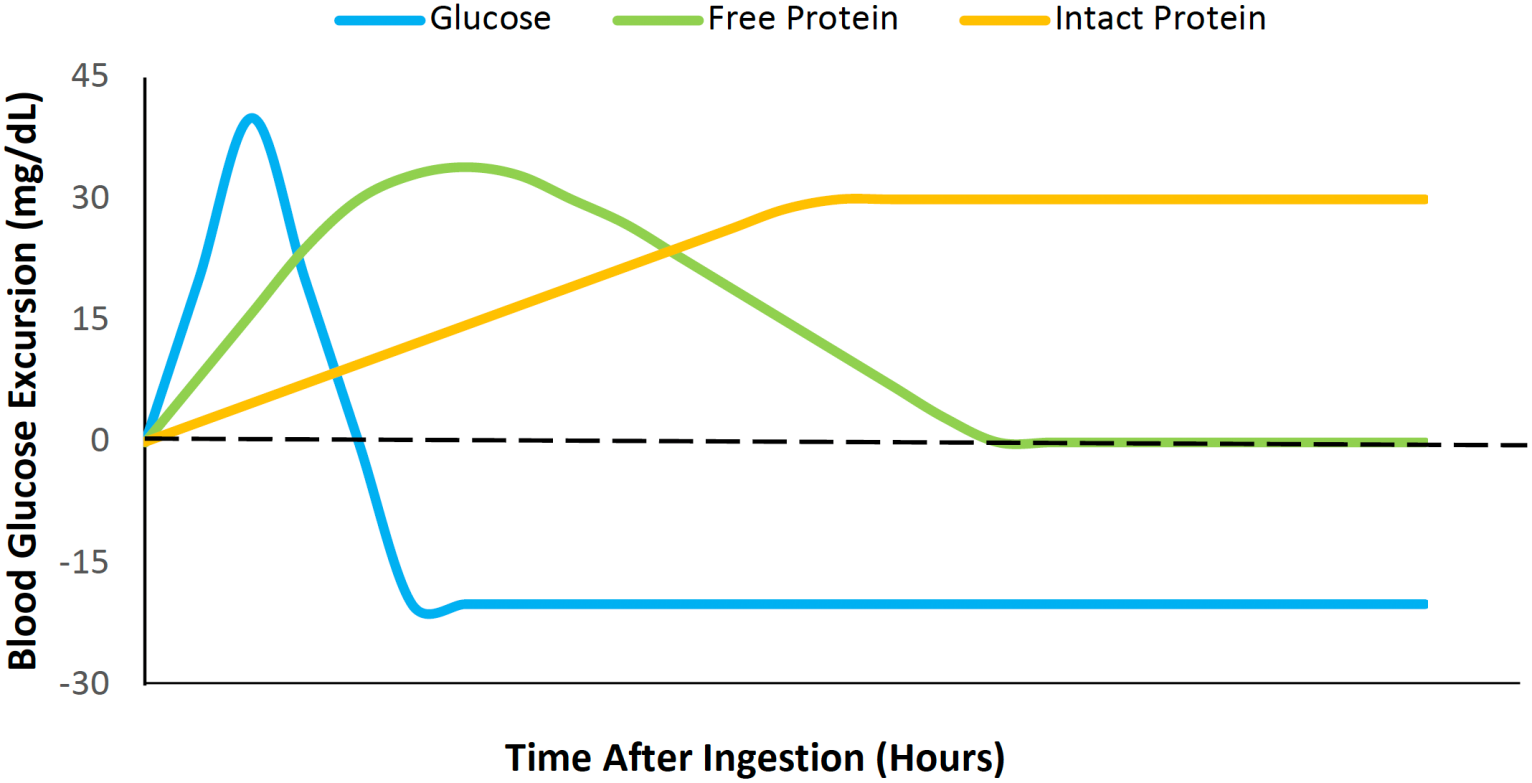
The effects of dietary intact protein, free protein, and glucose on blood glucose levels for 12 hours after ingestion, relative to baseline glycemia.

Compared to dietary carbohydrate, protein ingestion generally promotes glycemic stability by causing a slower and sustained rise in blood glucose and glucagon levels (31, 36, 37). Dietary protein exists in three major forms: intact proteins, free amino acids, and hydrolyzed proteins. The structure and complexity of the protein determine the rate at which it will be digested and, in turn, the hormonal and postprandial glycemic response (**Figure 1**) (18).

Intact proteins are full polypeptide chains that exist in their complete, functional form in the same way they are found in the body and require enzymatic digestion before absorption. In contrast, free proteins are individual amino acids not bound in a peptide chain, while protein hydrolysates are polypeptides that have been enzymatically cleaved into smaller peptides or free amino acids and are absorbed rapidly (38). Among these protein forms, intact proteins produce the most prolonged hormonal responses (insulin and glucagon) because they require digestion to release individual amino acids into the bloodstream, slowing down the rate at which the amino acids can stimulate the pancreas to release insulin and glucagon (36).

**Figure 2:**
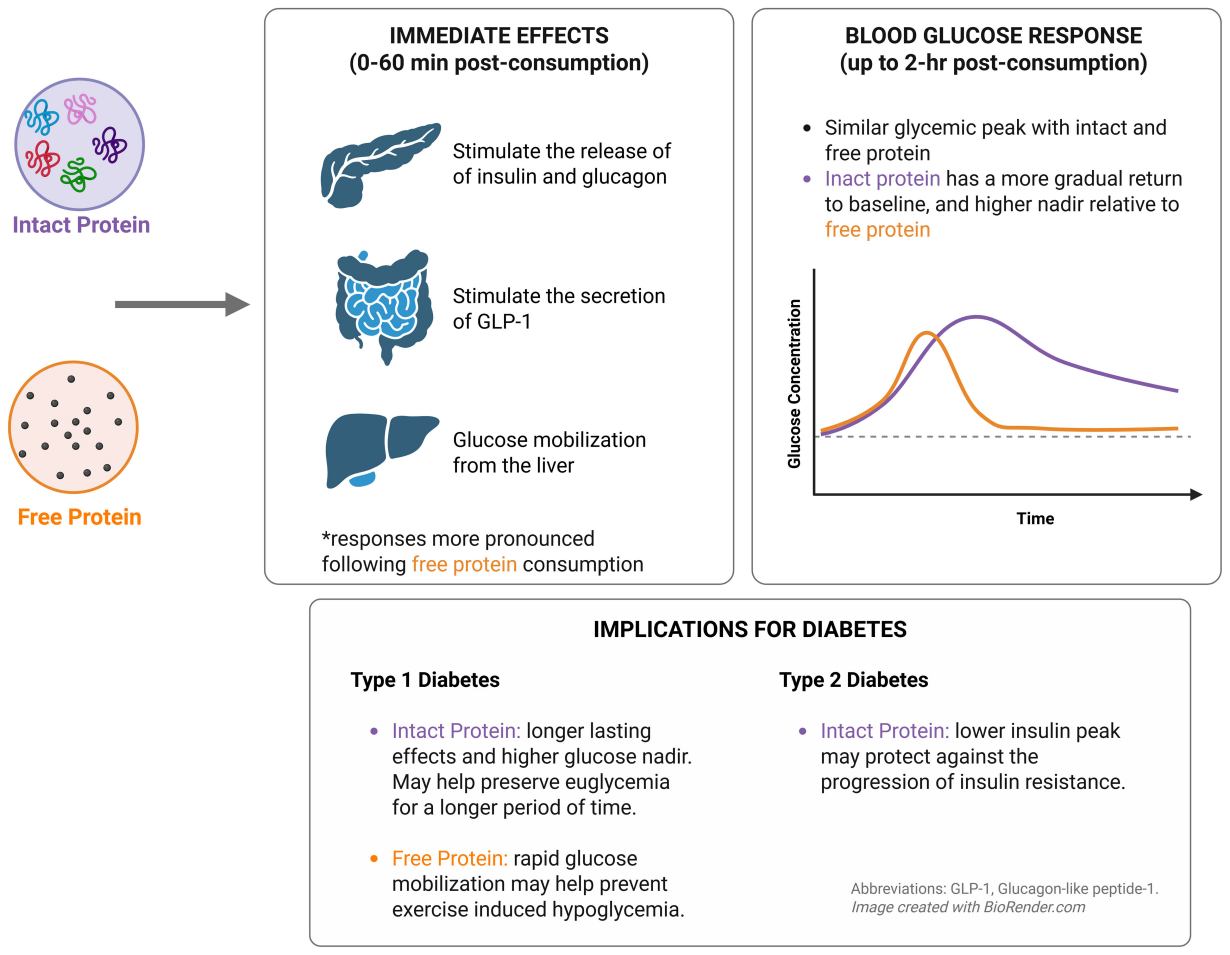
Summary of the glycemic and hormonal effects of intact versus free protein and the potential implications for each in diabetes management.

These differences in postprandial hormonal and glucose responses among protein forms have been observed in “healthy” (i.e., nondiabetic) rodent models. For example, in nondiabetic Wistar rats, higher levels of glucose, insulin, and glucagon-like peptide 1 (GLP-1) were observed 2 hours following the ingestion (via oral gavage) of a free amino acid mixture compared to intact albumin (25). While both treatments resulted in a similar glycemic peak (~160 mg/dL), intact protein induced a more gradual return to baseline glycemia. Two-hours after ingestion, blood glucose in the free amino acid group was significantly lower than the intact protein group (101.4 ± 5.0 mg/dL *vs* 123.7 ± 3.3 mg/dL; p<0.01), thereby suggesting that intact (whole) protein increases late-postprandial glycemia more than consuming free amino acids. A similar response has been observed in human studies. In individuals without diabetes who had recurrent postprandial hypoglycemia, a carbohydrate-reduced, high intact protein meal resulted in a lower peak blood glucose and a higher nadir blood glucose when compared to a standard meal (28). The meal that was enriched with intact proteins also produced comparatively higher glucagon levels and lower insulin, GLP-1, and glucose-dependent insulinotropic polypeptide (GIP) levels. Moreover, in one study of 12 healthy male adults without diabetes, intact soya protein significantly elevated the two-hour area under the curve (AUC) of both insulin (p=0.018) and glucagon (p<0.001) compared to hydrolyzed soya protein (18). Taken together, these studies highlight that intact protein may offer a more gradual and sustained hormonal effect and glycemic response, whereas free and hydrolyzed forms elicit a more immediate hormonal release but less glycemic stability over time. The distinct effects of each protein type have their own relevance in the strategic management of glycemia in T2D and T1D (**Figure 2**).

*T2D:*

Individuals with T2D and their caregivers may benefit from understanding the different glycemic responses to intact versus free protein. The lower peak insulin levels yielded by intact protein compared to free amino acids (25) may help delay the progression of insulin resistance, thereby benefiting those with T2D or prediabetes. On the other hand, the delay in peak insulin response may also negatively affect immediate postprandial glycemia, at least in theory, if carbohydrates are also consumed at the same time since the whole protein intake may delay insulin secretion.

Research on the impact of peptide complexity on postprandial glycemia in the T2D population is scarce. One study by Mortensen et al. (39) assessed the impact of four isocaloric mixed meals on the postprandial insulin response in T2D. The protein content of each meal was derived from either whey isolate (the intact form), whey hydrolysate, α-lactalbumin enhanced whey, or casein glycomacropeptide enhanced whey. The intact and hydrolyzed meals yielded a higher insulin 480 min glucose AUC compared to the two enhanced meals (p<0.001) but were not significantly different from each other (p>0.05). Peak insulin level was higher, however, with the hydrolyzed protein meal than with the intact protein meal (p<0.05). No AUC differences were seen for glucose, glucagon, GIP, or GLP-1 in that study (39). It should be noted that whey is a rapidly digested protein and there is evidence that, unlike other proteins, minimal differences in glycemic and insulin responses are seen between intact whey and its hydrolysate form (40, 41).

Another study investigated the interstitial glucose (i.e., using continuous glucose monitoring [CGM]) levels of overweight and obese nondiabetic individuals in response to a 12 gram serving of either intact casein protein or two different casein hydrolysates (i.e., A *vs*. B) (42). When consumed at breakfast, casein hydrolysate “B” showed a reduced postprandial glucose response compared to intact caseinate (p=0.039). No differences were found between hydrolysate “A” and either of the other groups, or between the three protein types when consumed in the evening. Despite the significance, further examination revealed that only 3 out of 18 individuals were classified as “responders,” meaning 15 out of 18 subjects did not experience a reduced glucose excursion following casein hydrolysate or intact casein (p<0.05) (42). The unexpected results may be attributed to the protein serving of 12 grams being too little to invoke a glycemic response when consumed on its own (23). The authors concluded that interindividual responses to protein intake may be highly variable, highlighting the need for “precision nutrition” (42).

In summary, the hormonal and glycemic response to intact and hydrolyzed protein within the T2D and overweight/obese population appears to be distinct from the healthy population and highly variable. More research exploring the postprandial insulin, glucagon, and glycemic response specific to T2D using a variety of protein types and doses is needed.

*T1D:*

In individuals with T1D, since the protein cannot stimulate endogenous insulin secretion as insulin is administered exogenously, the form of dietary protein may instead influence postprandial glucose dynamics by augmenting endogenous glucagon secretion. Notably, the sustained elevation in blood glucose levels caused by intact proteins may offer therapeutic benefits in situations of increased hypoglycemia risk, such as during prolonged fasting, overnight periods, or during and after exercise. In contrast, free or hydrolyzed proteins may act as a mild rescue strategy for hypoglycemia, with or without oral carbohydrates, due to their rapid absorption and potent glucagon-stimulating effects. On the other hand, and similar to in T2D, excessive protein intake a mealtime may increase late post prandial glycemia above the glycemic target range unless more insulin is administered.

Several studies have demonstrated a sustained glycemic effect following the ingestion of intact protein in individuals with T1D either alone or with carbohydrates and fat (22, 29, 43, 44). In a study by Smart et al. (44), children aged 8–17 with T1D consumed 4 breakfasts on 4 days all with the same amount of carbohydrate, but varying amounts of protein and fat. The protein portion of the meals were derived from eggs, intact whey protein, and milk. Regardless of fat content, high protein meals yielded higher blood glucose levels from 180 minutes post-ingestion onwards and reduced the risk of hypoglycemia (44). Thus, a breakfast that is rich in intact proteins may help to preserve euglycemia in T1D without promoting hyper- or hypoglycemia throughout the day. In a similar way, a high protein evening meal may help protect individuals with T1D from overnight hypoglycemia, but excessive consumption of protein might contribute to nocturnal hyperglycemia unless the prandial insulin dose is titrated accordingly. Supporting this, in a randomized crossover study, a high protein evening meal (110 grams of protein, 70 grams of carbohydrate, 52 grams of fat) significantly increased 12-hour glucose AUC by ~500 mg/dL*12h (27.8 mmol/L*12h) and glucose concentration 12 hours later by 62 mg/dL (3.4 mmol/L) compared to a standard evening meal in 15 adolescents with T1D on insulin pump therapy (29). These studies suggest that eating a meal with some intact proteins (~40-100 grams) may be a viable way for individuals with T1D to maintain euglycemia and/or reduce their risk of hypoglycemia, for several hours after a protein-rich meal.

Beyond prolonged glycemic elevations observed with intact protein, the fast action of free or hydrolyzed protein may make it an effective rescue or immediate preventative therapy from mild hypoglycemia in T1D (31, 33). During a sustained 40-minute hypoglycemic clamp, Rossetti et al. (33) showed that a 42-gram oral free amino acid mixture elevated glucagon levels during hypoglycemia as compared to placebo in individuals with and without T1D. Although glucagon levels were higher in the subjects without diabetes, this was attributed to the lack of glucagon counterregulation to hypoglycemia in the T1D group, not to any response variation to the amino acids. Notably, T1D participants receiving amino acids still had a significant elevation in peak glucagon levels (124 ± 25 ng/L) compared to placebo (49 ± 9 ng/L), thereby confirming that individuals with T1D can have a robust glucagon response to protein intake even if their glucagon counterregulatory response to hypoglycemia is blunted (33). Similarly, when 20g or 40g of oral alanine were provided after insulin induced hypoglycemia was achieved via a bolus subcutaneous injection, individuals with T1D displayed a reduced glucagon response relative to healthy controls but still had a significant and dose-dependent increase in both peak plasma glucagon and glucose (31). It should be noted that protein and/or carbohydrate sources also high in protein can increase insulin secretion, and should not be used to treat hypoglycemia in situations where endogenous insulin secretion is preserved, such as early post-diagnosis in T1D during the so-called “honeymoon” phase when endogenous insulin secretion still occurs (45).

## 3 Effects of protein type (plant *vs* animal) on glycemic response

The metabolic and hormonal effects of dietary protein are influenced not only by the form in which it is consumed, but also by its source (i.e., plant versus animal). The responses to protein type are distinct, largely due to differences in their amino acid compositions and rates of digestion (18, 19, 46, 47). Plant proteins are rich in arginine, a potent glucagon secretagogue (48), whereas animal proteins are typically rich in lysine and branched-chain amino acids (BCAAs, including leucine, isoleucine, and valine) which are strong stimulators of insulin secretion (19, 36).

In general, plant proteins are considered slow-digesting, while animal proteins are considered fast-digesting. Typically, proteins that are digested more rapidly induce a greater glucose excursion and a more immediate insulin and glucagon response (49). Exceptions to this generalization, including casein (a slow-digesting animal protein) and pea (digests faster than other plant proteins), suggest that digestion rate should also be considered when looking at glycemic responses (26, 47).

*T2D:*

In individuals with T2D, where insulin secretion is often blunted and insulin resistance is elevated, the choice of protein both in terms of source (animal *vs*. plant) and digestive rate (fast *vs*. slow) may be important in postprandial responses. Several clinical trials have investigated whether replacing animal-derived proteins with plant-based options may improve glycemic outcomes in T2D (26, 27, 47, 50). One of the most comprehensive evaluations comes from a systematic review and meta-analysis of 13 randomized controlled trials by Viguiliouk et al. (27), which found that replacing a median of 35% of total protein per day with plant protein for a median duration of eight weeks significantly lowered HbA1c level (mean difference -0.15%, p=0.009), fasting glucose (mean difference -0.34 mmol/L, p=0.02), and fasting insulin level (mean difference -10.09 pmol/L, p=0.006) in individuals with T2D, suggesting that that plant protein substitution may help mitigate hyperglycemia and hyperinsulinemia in this population. An additional meta-analysis found that the risk for T2D increased with increasing consumption of total protein and/or animal protein, while plant protein and yogurt had an inverse relationship (i.e., they were associated with reduced T2D risk) (50). Taken together, sticking to a moderate, rather than high protein diet and opting for plant based protein sources may help to limit one’s risk for developing T2D, and/or help those with T2D better manage hyperglycemia (27, 50).

While both of the above meta-analyses (27, 50) attributed the observed glycemic benefits to opting for proteins of a plant origin, the difference may actually stem from increasing the ratio of slow digesting proteins to fast digesting proteins that makeup the diet. Typically, plant proteins are considered slow-digesting, whereas animal proteins are comparatively fast-digesting, however there are exceptions. For example, following a six-week diet of either pea (medium-fast digesting plant protein) or casein (slow digesting animal protein), the casein group was associated with improved insulin sensitivity and secretion, as well as a lower 4-hour AUC of insulin (p=0.023) and glucagon (p=0.015) following a post-intervention meal-tolerance test (47). This benefit could be attributed to pea protein’s faster digestion rate, which may have led to a sharper glucagon rise and thus greater insulin demands. Unlike the results observed by Viguiliouk et al. (27) this study found that the animal protein was associated with lower insulin and glucagon levels. We believe that this incongruency indicates that a protein’s rate of digestion is a stronger indicator of its glycemic response than its origin. Supporting this, one study in adults with T2D comparing the glycemic response to two animal-based proteins, whey (fast-digesting) and casein (slow-digesting), found that the faster-digesting whey protein stimulated a greater insulin peak (301.4 pM *vs*. 215.3 pM, p<0.015) and 180 min AUC (33.2 nU/mL*180 min *vs*. 26.6 nU/mL*180 min, p<0.05) (26).

The relationship between protein type, digestion rate, and glycemic effect is not always straightforward. These studies (26, 27, 47, 50), suggest that diets emphasizing slower-digesting proteins, regardless of origin, may be more effective in modulating postprandial glycemia in T2D and/or prediabetes.

Beyond digestion rate, branch chain amino acids (BCAA) content also warrants careful consideration in relation to T2D management. BCAAs are commonly found in animal protein sources, especially meat and dairy. BCAAs have a strong effect on pancreatic beta cells, and as such, overconsumption of these amino acids can cause chronically elevated insulin levels (51, 52). In people without diabetes, higher plasma levels of BCAAs are associated with lower insulin sensitivity, reduced metabolic clearance of insulin, and higher fasting insulin concentrations (53), thereby linking high BCAA intake, potentially, to the development of insulin resistance, especially when paired with obesity (54). Indeed, studies have consistently highlighted that individuals with T2D have higher fasting plasma BCAAs compared to individuals without diabetes (55, 56). However, some researchers argue that it is the phenotype of insulin resistance that causes dysregulated BCAA metabolism, while others propose the contrary, that high consumption of BCAAs is the driver of insulin resistance (57). Currently, there are no guidelines for BCAA consumption in diabetes. Regardless of directionality, more research into the specific effects of a BCAA rich diet in T2D management and/or progression is required to help inform proper recommendations for consumption. The rise in incretin therapies for weight loss, glycemic management and organ health, often with therapy-related sarcopenia (58), also warrants further investigations on if certain protein supplements may be useful for offsetting the loss in muscle mass without resulting in protein-mediated insulin resistance.

In summary, in T2D, the type of protein consumed has important implications for the release of glycemic hormones, especially insulin. Fast-digesting proteins and proteins rich in BCAAs stimulate insulin more potently than their counterparts, which in the long run may increase insulin resistance. If protein supplementation helps preserve muscle mass when incretin therapy is initiated is currently unknown.

*T1D:*

In the same meta-analysis as above (27), trends in T1D were similar, yet did not achieve statistical significance. It was suggested that replacing a median of 35% of total protein per day with plant protein for a median duration of eight weeks could be associated with a lower HbA1c level in T1D (mean difference -0.64%, p=0.11) and lower fasting glucose (mean difference -1.78 mmol/L, p=0.13) (27). The results may have been insignificant because the researchers only found two studies (59, 60), highlighting the need for more research on the impact of plant and animal protein in the T1D population.

**Figure 3:**
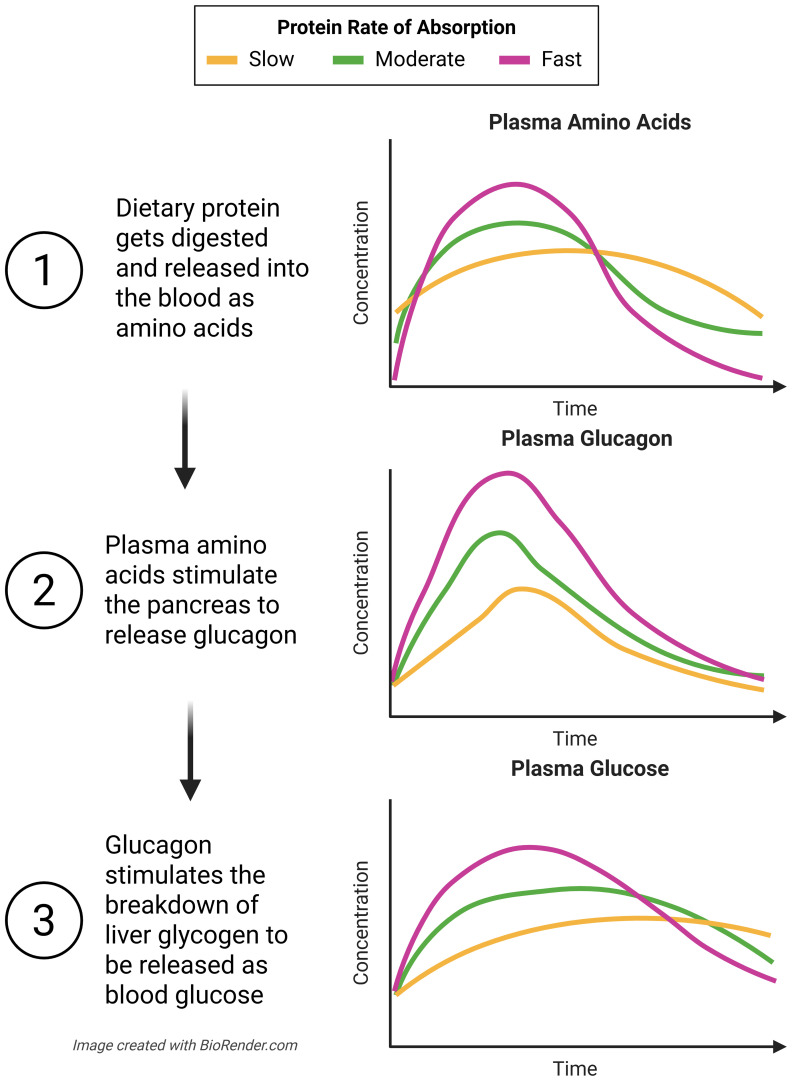
A protein’s absorption rate indicates the rate of amino acid, glucagon, and glucose appearance in the blood. Faster absorbing proteins yield a faster and stronger postprandial endocrine and glycemic response than slower digesting proteins. Adapted from (32).

As with T2D, it is likely the differences observed between plant and animal protein on HbA1c and fasting glucose in T1D are related to their respective rate of digestion. The glucagon, and subsequent glucose response to dietary protein is linearly related to changes in plasma amino acid levels (36). Thus, faster absorbing proteins evoke a sharper glycemic response than slower absorbing proteins because the former promotes a greater glucagon rise than the later (32) (**Figure 3**).

In a previous section, we discussed how peptide complexity (i.e. intact, hydrolyzed, or free protein) affects the postprandial endocrine and glycemic response. Longer peptide chains take longer to digest because they require enzymatic breakdown into free amino acids (36). As such, the relationship between slow *vs* fast digesting protein is similar to that of intact *vs* free protein, which is described above. Other factors that influence a protein’s absorption time include food processing (61) and other nutritional components of the meal, namely fat (62) and fiber (63) content (**Table 1**). Individuals with T1D may be able to take advantage of various protein absorption rates to manage their glycemia. For example, slow proteins (such as casein, pea, whole eggs, meat/poultry etc.) could be used to minimize one’s risk of hypoglycemia, similar to previously described implications for intact proteins, and fast proteins (such as whey, collagen, egg whites, lean fish etc.) may have potential as a rescue from mild hypoglycemia and/or as a pre-exercise intervention against hypoglycemia. However, to our knowledge, no studies have looked specifically into any of these potential implications.

**Table 1:** Factors that affect a protein’s rate of absorption.

Factor that influences absorption rate	Slower absorbing	Faster absorbing	Reference
Peptide length	Intact protein	Protein hydrolysates and free amino acids	(36)
Food processing	Unprocessed	Exposure to high temperatures, extreme pH, mechanical breakdown	(61)
Other nutritional components	Addition of fat and/or fibre	Protein alone	(62, 63)
Solubility	Insoluble in water	Soluble in water	(64)
Coagulation in the stomach	Coagulates (i.e. casein)	Does not coagulate (i.e. whey)	(61)
State	Gelled or solid	Liquid	(65)

Modifying these factors can change the glycemic response to protein ingestion in type 1 diabetes (T1D) and type 2 diabetes (T2D).

A protein’s absorption rate is one of many factors that influence the postprandial endocrine and glycemic responses. Since this is complicated by the interactions of many properties of protein, Dao et al. (32) suggest the need for an aminoglucogenic index to help individuals with T1D understand how various protein foods will impact their blood glucose levels. Similar to how the glycemic index ranks carbohydrate sources based on how quickly they induce a rise in blood glucose post ingestion, an aminoglucogenic index would rank protein sources on the rate by which amino acids appear in the bloodstream after ingestion, and subsequently affect blood glucose levels. As such, the index could be used to help influence food choices and/or exogenous insulin dosing strategies to ultimately improve postprandial TIR for individuals with T1D. We propose that this index could be beneficial in a mobile app format which would allow users to select foods that they are eating, and view the projected blood glucose excursions and/or historical glucose responses to such foods. This information could also be integrated into insulin delivery decision-making tools (i.e., how much more prandial insulin is needed because of protein intake, and when).

Before an aminoglucogenic index could be developed however, several foundational steps would need to be established. Research is needed to establish the glycemic response to various protein sources, both alone and in combination with carbohydrates and fats at various ratios. To our knowledge, no studies have examined whether an identical protein-containing meal would elicit a similar glycemic response within and between individuals. Finally, once established, the index would need to be widely validated on a large number of individuals with T1D using CGM to assess if its application could meaningfully improve glycemia. Overall, more research is needed into the potential clinical implications of protein source and absorption rate in T1D.

## 4 Protein with or without carbohydrate co-ingestion

**Figure 4:**
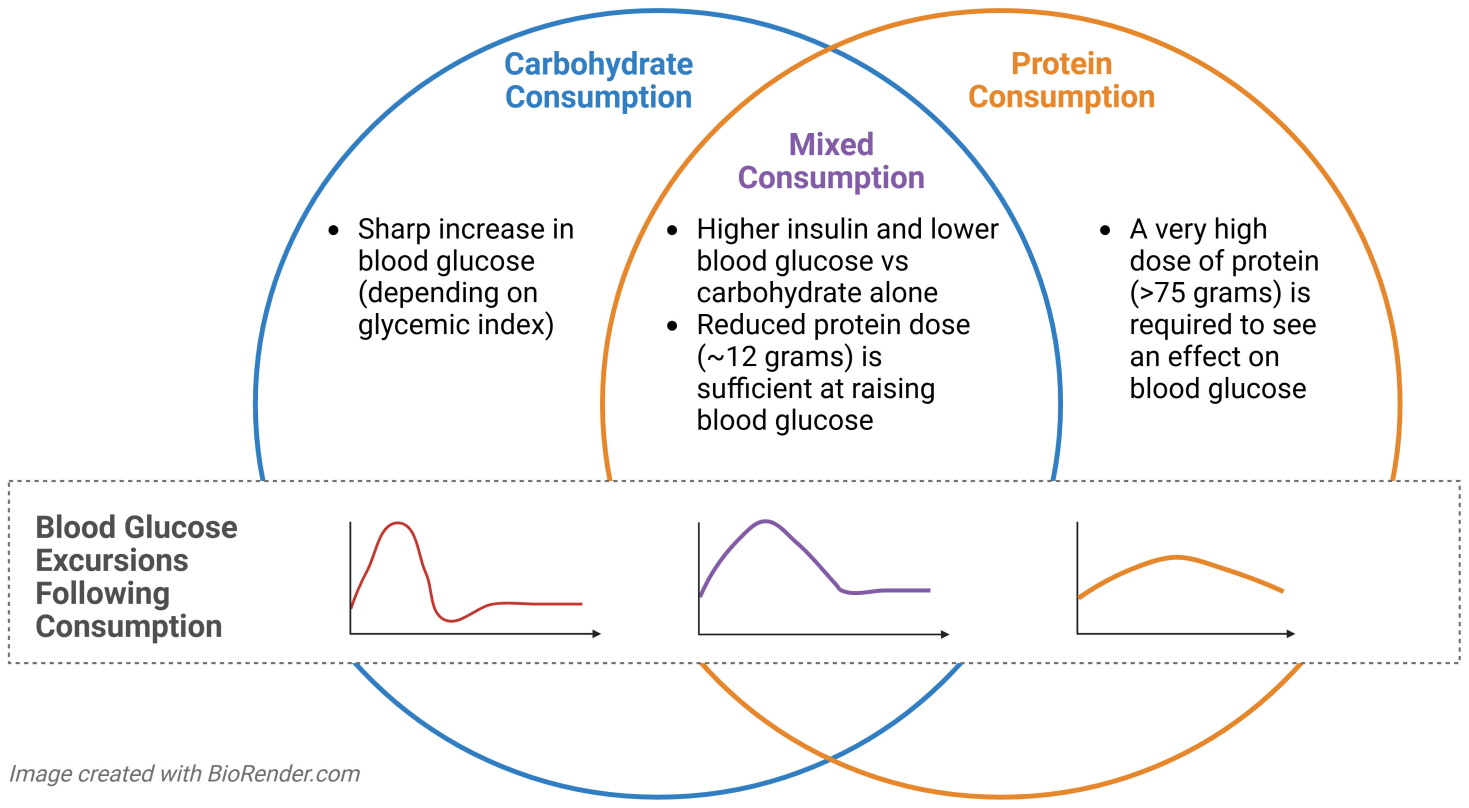
Summary of the response to carbohydrate and protein ingestion in isolation or combined in a mixed meal.

Protein ingestion in isolation or in combination with carbohydrate exerts different effects on glycemic hormones and responses (**Figure 4**). In the absence of any other dietary macromolecules, amino acids stimulate both glucagon and insulin secretion (37), although the ratio of hormone secretion is dependent on the protein dose (18).

At a higher dose of protein in isolation, at least in healthy (nondiabetic) populations, amino acids tend to preferentially stimulate glucagon secretion over insulin secretion, resulting in a lower insulin-to-glucagon ratio, and a higher blood glucose level. On the other hand, when protein consumption is combined with carbohydrate, it markedly attenuates the carbohydrate-induced rise in plasma glucose. Blood glucose levels are typically lower following protein and carbohydrate co-ingestion compared to carbohydrate consumption alone (20), and this is partially attributed to glucose’s ability to suppress amino acid-stimulated glucagon release through non-competitive inhibition (37). Simultaneously, insulin secretion is elevated following carbohydrate and protein co-ingestion versus carbohydrates alone (66). The dual action of reduced glucagon and increased insulin secretion makes protein a potential modulator of the postprandial glucose when it is co-ingested with carbohydrate.

*T2D:*

In T2D, insulin resistance and impaired insulin secretion exist, and while protein can stimulate insulin secretion, the efficacy of that insulin in lowering blood glucose can be blunted. Acute studies have demonstrated that the co-ingestion of protein and carbohydrate can markedly increase the insulin response in individuals with T2D compared to carbohydrate alone (66). In adult males with T2D, consumption of carbohydrate with a free amino acid mixture increased insulin responses by 189% compared to carbohydrate alone. There was no difference, however, in plasma glucagon or glucose levels (p>0.05) between trials, indicating that the subjects had little or no sensitivity to insulin. These findings highlight the potential benefit of protein-induced insulin stimulation in individuals using insulin-sensitizing medications like metformin, but also underscore its limited utility in those with severe insulin resistance.

*T1D:*

In T1D, the glycemic effects of protein intake are influenced by the dose and type of protein, and if the protein is co-ingested with other macronutrients. For example, in the absence of other macronutrients, a greater dose of protein is required in individuals with T1D to see the same glycemic effect as when a mixed meal is consumed (23, 24). One study by Paterson et al. (23) reported no change in 5-hour blood glucose levels when adults with T1D consumed 8 drinks (randomized over 8 days) containing either 0, 12.5, 25, 50, 75, or 100 grams of whey protein isolate or 10 or 20 grams of glucose with no insulin, until the pure protein dose reached 75 grams. Compared to the 20 grams of glucose drink, 75 or 100 grams of protein yielded significantly delayed and sustained blood glucose excursions of ~72 mg/dL above baseline, but the doses of 50 grams or below had no effect. In a follow-up study, the team conducted a similar protocol but with the addition of 30 grams of carbohydrate to each protein drink (24). From 150 min onward, all protein doses resulted in a higher blood glucose level than control (i.e., carbohydrate only), in a dose dependent manner. These studies suggest that in the absence of other macromolecules, a very high dose of protein (≥75 grams) is needed to invoke a clinically significant rise in blood glucose level in T1D. However, when combined with carbohydrate, a dose of protein as low as 12.5 grams may raise blood glucose to a similar extent, or even higher, than carbohydrate alone (23, 24). This protein-induced glycemic effect has practical implications for meal planning and exercise-related glucose management in T1D, as further discussed in the next section.

## 5 Effects of protein dose and timing on glycemic response

**Figure 5:**
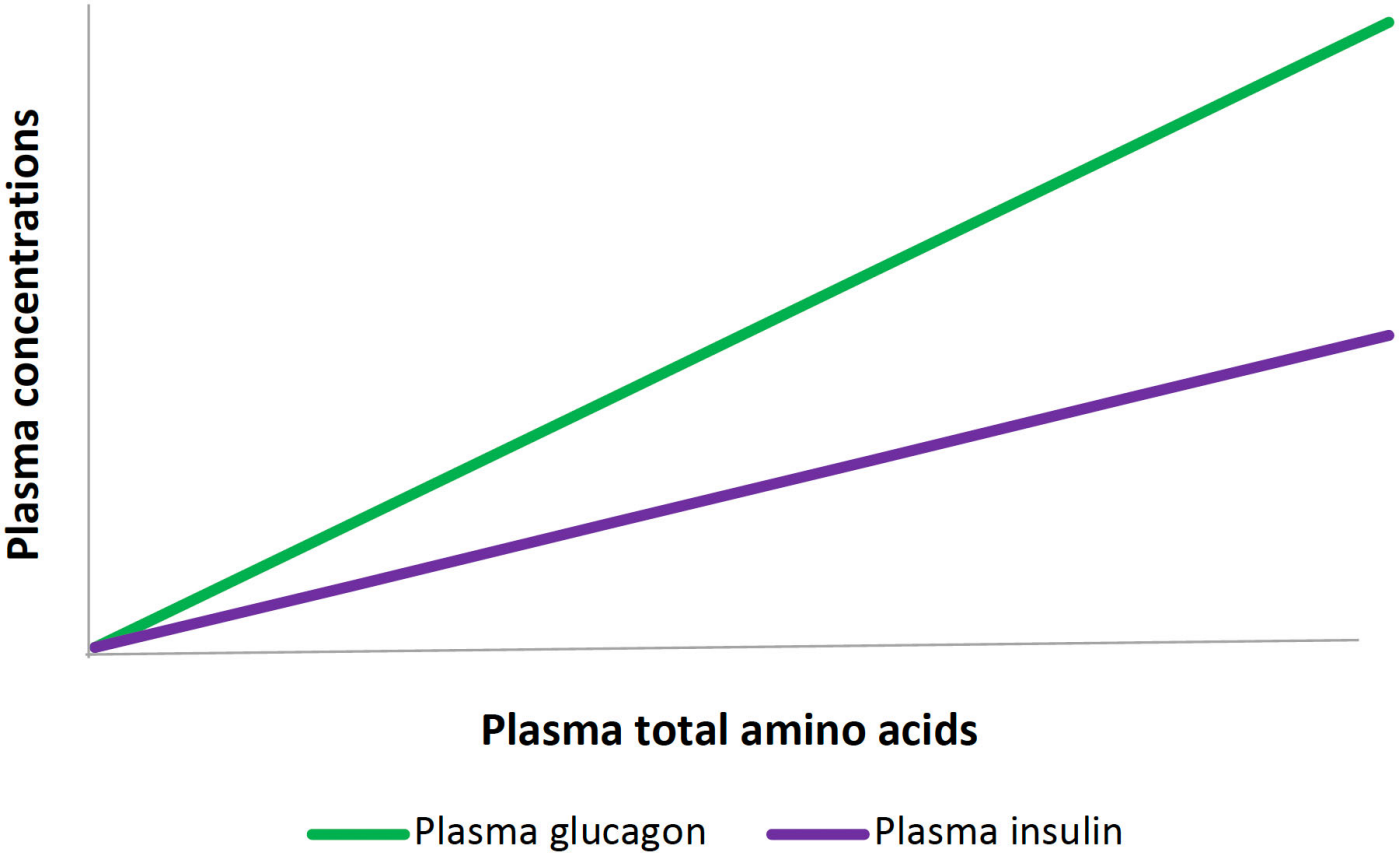
Plasma amino acids stimulate both insulin and glucagon secretion. A higher concentration of plasma amino acids stimulates glucagon more potently than insulin. As a result, higher doses of protein lead to a higher glucagon: insulin ratio.

Due to the unique glycemic and hormonal response to protein, proper timing of protein intake can help mitigate dysglycemia (i.e., hypoglycemia, hyperglycemia) and hyperinsulinemia. Similarly, improper timing of protein can exacerbate these issues. Protein dose-dependently stimulates pancreatic alpha-cells (to secrete glucagon) more potently than beta-cells (to secrete insulin), meaning a higher dose of protein will result in a higher glucagon-to-insulin ratio (**Figure 5**) (18). Over the long term, habitual protein intake may also influence overall glucose metabolism. When comparisons were made between people who maintain a normal protein diet (0.57-0.80g/kg/day) or a high protein diet (1.25-2.41g/kg/day) for at least six months, then subjected to an intravenous glucose tolerance test and a euglycemic hyperinsulinemic clamp, the high protein group had significantly increased ability to release insulin and glucagon, as well as a 12% higher endogenous glucose output when at a low plasma insulin concentration (~40 pmol/L, p<0.01) (11). Careful timing of protein ingestion, and consideration of the dose consumed, have implications for preserving insulin sensitivity and reducing hypoglycemia during and after exercise.

*T2D:*

Due to the hyperglycemic phenotype of T2D, protein stimulated endogenous insulin secretion may be seen as an acutely beneficial way to minimize postprandial hyperglycemia in those with prediabetes or T2D (67). However, a chronically high protein diet could be detrimental for those with prediabetes or T2D (68).

As noted above, protein intake stimulates both insulin and glucagon secretion (18), and a high protein diet is associated with elevated insulin and glucagon in T2D individuals (11). This metabolic profile of hyperglucagonemia and hyperinsulinemia for a prolonged period can exacerbate the T2D phenotype and progresses the disease’s severity (69, 70).

Hyperinsulinemia and insulin resistance go hand-in-hand, although it is debated which one causes the other. Some researchers propose that insulin receptors become desensitized first, prompting the body to secrete more insulin in compensation (71). Others argue that hyperinsulinemia results from overnutrition, and tissue desensitization develops as a compensatory response to protect the tissues and prevent hypoglycemia (72). Nonetheless, numerous studies have found connections between long-term high protein intake, insulin resistance, and T2D risk (73–76). Long-term excessive protein induced hyperinsulinemia is linked with peripheral insulin resistance.

In a similar manner, chronic high protein diets may contribute to hepatic insulin resistance via a sustained hyperglucagonemia. Glucagon is secreted from the alpha cells of the pancreas into the portal vein, meaning the liver sees the highest concentration of glucagon in the bloodstream. Elevation in fasting glucagon concentrations are associated with non-alcoholic fatty liver disease (77). Hyperglucagonemia also prompts excessive gluconeogenesis, which contributes to hepatic insulin resistance (78).

For these reasons, a chronic high protein diet may not be suitable for those with T2D or prediabetes, as excess amino acids can desensitize tissue to insulin (79) and contribute to other organ complications. Thus, daily protein intake should be kept within a moderate range (0.57-0.80g/kg/day) for these populations (11).

**Figure 6:**
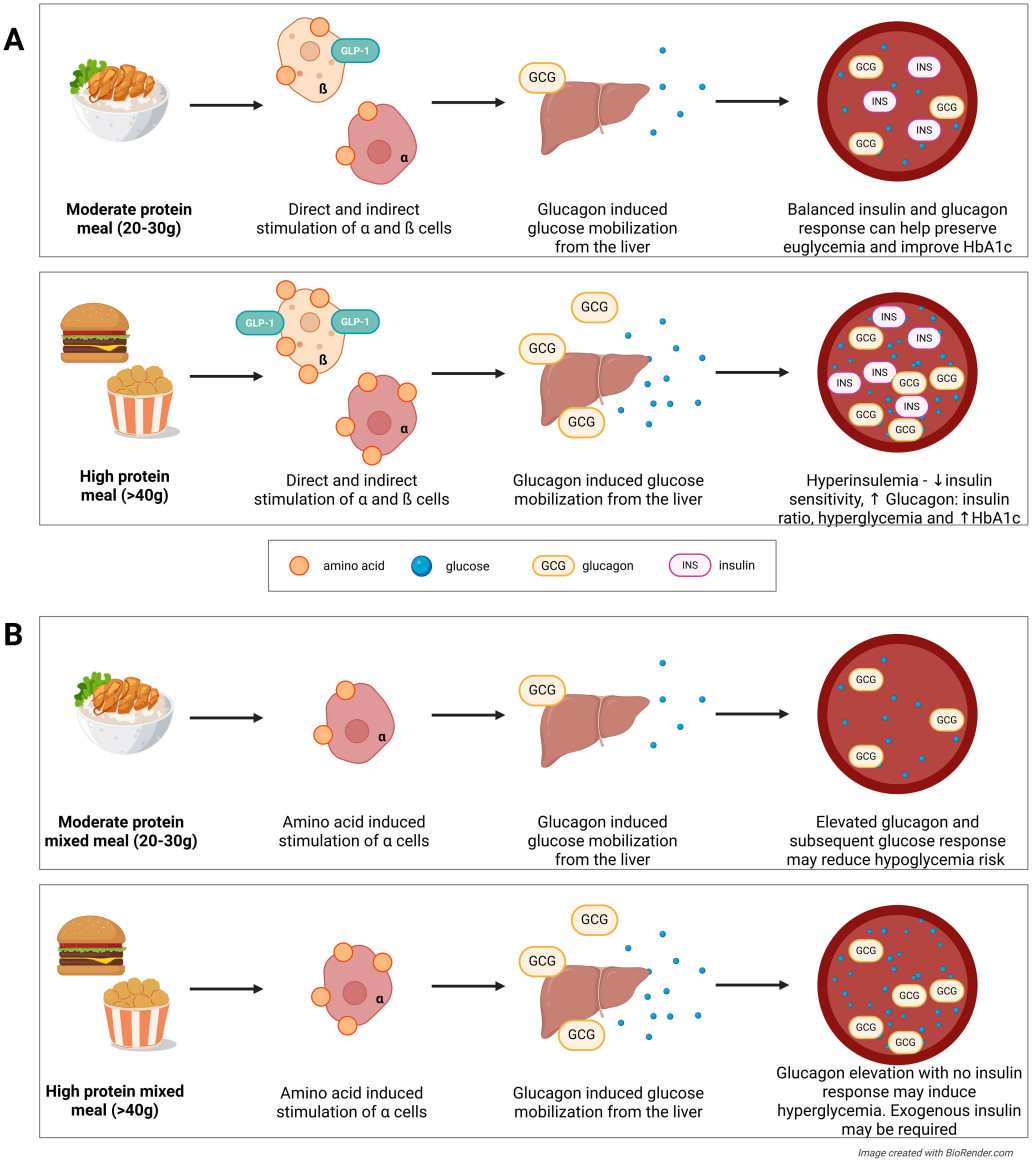
**(A)** The acute effects of a high protein meal in type 2 diabetes (T2D) include hyperinsulinemia and an elevated insulin: glucagon ratio. Chronic exposure can lead to insulin resistance and elevated HbA1c, respectively. **(B)** In type 1 diabetes (T1D), a high protein meal evokes an unopposed glucagon response which in turn, elevates blood glucose levels. This could have implications for hypoglycemia prevention, but could also result in postpriandial hyperglycemia.

In addition to monitoring overall total daily protein intake, the timing of protein intake within a day should be considered. High protein meals invoke postprandial hyperinsulinemia which can depress insulin sensitivity if repeated frequently (80). This has been associated with worsened glycemic outcomes (as measured by elevated HbA1c) in T2D (81) (**Figure 6A**). The above-mentioned negative effects of a high protein diet (hyperinsulinemia, hyperglucagonemia, and their associated effects) are also displayed acutely from a single high protein meal, posing the same risks as a high protein diet, albeit to a lesser extent. For optimal management, those with prediabetes and T2D should likely maintain a moderate protein diet and ensure that their protein consumption is evenly spaced throughout the day, avoiding high protein meals.

More research is needed to understand the effects of protein intake timing and frequency in relation to meals and daily patterns in this population.

*T1D:*

In T1D, protein timing may play a unique role in managing hypoglycemia, particularly around exercise. While carbohydrates are the current recommended strategy to prevent and treat low blood glucose in those with T1D, their rapid but short-lived glycemic effect often requires frequent re-treatment, especially during prolonged activity.

Emerging evidence suggests that pre-exercise protein intake may improve CGM TIR, reduce time below range (TBR: <70 mg/dL) and reduce the necessity for frequent intervention to maintain euglycemia during exercise in this population (82, 83). In adolescents with T1D, consumption of 10 or more grams of protein within the 4 hours prior to a bout of moderate to vigorous physical activity was associated with reduced TBR by 5.38% with a dose of 0.125-0.249g/kg (p=0.01) and 4.32% with a dose of ≥ 0.25 g/kg (p=0.03) compared to those who did not consume protein (83). Although research on this topic is still limited, the findings from the mentioned study (83) raise important questions about the potential benefits of pre-exercise protein, particularly in forms that digest quickly, such as free amino acids or hydrolysates as potential tools to mitigate exercise-induced hypoglycemia.

To our knowledge, studies have yet to be published on the effects of pre-exercise free proteins or protein hydrolysate on the prevention of exercise induced hypoglycemia in T1D, although work on this topic has been presented at recent scientific conferences (84). These protein forms are known to produce a glucose response, likely by increasing glucagon secretion, that is relatively quick and moderately sustained (36, 37), making them a possible pre-exercise tools when exercise-induced hypoglycemia is a known threat, or in instances where continuous fueling (e.g., with glucose) during exercise is impractical, such as during competition or extended activity without breaks. Moreover, since the glycemic response to protein intake can be longer acting than that of simple carbohydrate ingestion or even exogenous glucagon administration (31, 36, 37), it is possible that including oral protein in a hypoglycemic rescue snack/meal could help prevent hypoglycemic rebound.

Protein timing may also play a role in the prevention of delayed, post-exercise hypoglycemia. In one euglycemic clamp study, adolescents with T1D consumed either water or a drink containing 50 grams of whey protein isolate 3.25 hours after a late-afternoon (4:00PM) moderate intensity exercise session (22). Compared to whey protein, the mean glucose infusion rate to maintain euglycemia was six-fold and 2.5-fold higher during the 4 hour late-onset post-exercise hypoglycemia risk and overnight periods, respectively, with water-only (22). These findings suggests that consuming protein in the evening after exercise can help reduce the risk of late-onset hypoglycemia. While potentially promising, more randomized controlled trials are needed to evaluate the timing of protein consumption relative to different exercise session types, intensities, and durations.

## 6 Other considerations: the use of insulin delivery systems in T1D

Different modalities of insulin delivery are available for individuals living with T1D and include multiple daily insulin injections (MDI), standard insulin pumps, and automated insulin delivery (AID) systems. While standard insulin pumps deliver fixed basal rates set by the user, AID systems use CGM data to automatically adjust basal insulin delivery in response to glucose levels and trends to try and keep the individual within a predetermined glucose range. Regardless of insulin modality, individuals with T1D must administer mealtime bolus insulin to cover any carbohydrates consumed. This bolus dose is calculated based on the amount of carbohydrates in the meal and the user’s personal insulin: carbohydrate ratio, but does not account for any other macronutrients.

The bolus dose calculations in current AID systems do not account for the consumption of other macronutrients, and as previously discussed, the glucagon response following protein consumption is unopposed in T1D which can cause delayed hyperglycemia. For example, a high protein mixed meal tends to induce a greater glycemic excursion later in the post-absorptive period than an isocaloric low protein meal, thereby altering exogenous insulin requirements (**Figure 6B**). As such, a 20% increase in the insulin bolus dose calculated for carbohydrate alone is a commonly considered starting point that all individuals with T1D can implement if a significant portion of protein is consumed with carbohydrate-containing foods. However, as pointed out by various professional diabetes organizations, adjustment of insulin doses for protein (or fat) should ultimately be guided based on the individuals own glycemic patterns following meals, and only when there is evidence of a rather consistent postprandial glycemic response within the individual (85).

Moreover, more nuanced approaches to bolus dosing for high protein mixed meals in MDI and standard pump users have been evaluated in the literature (86, 87). For instance, a systematic review of eighteen studies including MDI and standard pump users found that an additional 24-75% increase in the patient’s insulin to carbohydrate ratio is beneficial for high fat and high protein meals (87). Standard pump users have the additional option to use a “dual-wave” or “square-wave” insulin bolus delivery pattern where only a portion of the total bolus insulin dose is delivered immediately (often 50–70%) and then the remainder is delivered slowly over an extended period of time (86). Smith et al. highlighted that a 60% upfront bolus dose, 15 minutes prior to the meal may be beneficial to reducing late post prandial hyperglycemia (87). However, inter-individual insulin requirements to maintain euglycemia with protein consumption are highly variable (88) and some “trial and error” may be required to individualize the insulin dose for high protein meals. The varying glycemic responses to protein types, doses, and relation to other macronutrients within a mixed meal further highlight the need for the development of a so-called “aminoglucogenic index,” as previously discussed, to help individuals with T1D make more informed insulin dosing decisions to help maintain glycemic TIR following high protein intake (32).

The major benefit of AID systems is their ability to automatically adjust basal insulin delivery in response to glycemic excursions (such as those following a high-protein meal), thereby reducing the users burden (89). However, the impact of dietary protein on automatic basal insulin adjustments by AID systems remains an active area of research, as current algorithms cannot fully account for the varied postprandial meal responses (90). For example, one study investigating the dietary determinants of postprandial glycemia in individuals with T1D using an AID system found that, at breakfast, the percent intake of total protein is a negative predictor of TIR (p<0.05) (91), whereas plant protein intake at dinner was a positive predictor of TIR (p<0.5). In that study, the AID basal insulin delivery involved a combination of pre-meal boluses, automated post-meal micro-boluses, and user-initiated correction boluses. The authors noted that meal composition imposes differing effects on postprandial blood glucose profile and insulin requirements, thus remaining an ongoing challenge to AID systems (91). Similarly, another study which compared AID systems found that the postprandial glucose response was similar between different pumps, despite differing AID algorithms and automatic insulin delivery patterns (92). More research is needed to refine AID algorithms so they more effectively account for dietary protein intake.

Protein type may further complicate AID system performance. Fast versus slow digesting proteins have distinct absorption profiles and may also necessitate different algorithm requirements to improve postprandial glycemia. Moreover, the appearance of amino acids in the bloodstream may not be an accurate predictor of future glycemia, because different amino acids are absorbed at different rates. AID systems cannot currently anticipate these dynamics unless they are informed of the meal composition, meaning some degree of user input will likely remain.

Exercise also presents an ongoing challenge for AID system users (89). AID systems require the user to manually reduce their basal and/or bolus insulin delivery or initiate a higher ‘temporary target’ 1–2 hours before they start exercise to help limit circulating basal insulin. If the user forgets to do this, or is not able to predict their exercise bout, the high circulating insulin poses a risk of hypoglycemia. The pre-exercise consumption of free amino acids or rapidly absorbed proteins (such as whey) could be beneficial for reducing the risk of exercise-induced hypoglycemia. By stimulating endogenous glucagon secretion, protein could be a useful ‘backup plan’ if the AID higher temporary targets were not implemented well in advance of activity and circulating insulin was elevated. However, fast acting carbohydrates should always be on hand for safety. Future studies should examine the effects of fast acting protein with and without carbohydrate co-ingestion on the blood glucose levels of exercising individuals with T1D utilizing AID systems.

Taken together, while advancements in insulin delivery technologies have improved glycemic management in T1D, protein’s complex and delayed metabolic effects continue to pose challenges across all insulin delivery modalities (MDI, standard pumps, and AID systems).

## 7 Discussion

### 7.1 Clinical implications for T1D, T2D, and prediabetes

Careful timing and selection of protein sources can help individuals with diabetes manage their glycemia and limit their complications. Since individuals with T1D do not secrete insulin, protein can be a useful way to boost glucagon on its own (22, 31, 33). This may have implications for the prevention of hypoglycemia, whether nocturnal or induced by exercise or fasting. A snack containing both carbohydrate and protein is an effective way to boost glucose levels for a sustained period (23, 24). Additionally, a high protein breakfast may be an effective way for individuals with T1D to maintain euglycemia throughout the day (28). It has also been shown that the glucagon response to protein is preserved when glucagon counterregulation to hypoglycemia is abolished, making free amino acids an effective rescue treatment to mild hypoglycemia (31, 33).

The effects of protein in T2D and prediabetes is more complex. Acutely, protein stimulates endogenous insulin release, which can be beneficial for lowering blood glucose levels in these populations (66). On the other hand, some proteins are stimulates of glucagon secretion which can increase hepatic glucose production thus resulting in both hyperinsulinemia and hyperglucagonemia. Moreover, the chronic elevation in circulating insulin which may accompany a long-term high protein diet, could further promote insulin resistance, worsening the diseases’ severity (35, 79). In addition, sustained glucagon secretion may result in liver dysfunction of protein and glucose turnover (93). For this reason, nutritional guidance aimed at reducing postprandial insulin spikes must consider protein intake, not just carbohydrate. Approaches such as choosing intact, slow-digesting proteins, moderating total protein load, and slowing digestion with added fat or fiber can help maintain smoother hormonal responses (11, 25, 26). The amino acid profile—including attention to BCAAs, which are potent insulin secretagogues—also plays a key role.

Across both conditions, the clinical effects of protein on post-meal glycemia depend on its form (intact *vs*. hydrolyzed *vs*. free amino acids), source and digestion rate (plant *vs*. animal; slow *vs*. fast), co-ingestion with carbohydrate, and dose and timing. Intact and slowly digested proteins generate a gradual rise in circulating amino acids, producing steadier glucagon and insulin release and more prolonged glycemic effects. In contrast, hydrolyzed proteins and free amino acids are absorbed rapidly, eliciting sharper hormonal responses and more abrupt glucose changes (18). These physiologic differences shape practical strategies: in T2D, slower proteins or diets emphasizing plant-based options can reduce insulin demand and help mitigate hyperglycemia over time, while fast-digesting proteins or BCAA-rich sources can acutely lower glucose but may exacerbate hyperinsulinemia if consumed excessively (11, 27, 57, 79). In T1D, where exogenous insulin is required, protein’s glucagon-stimulating properties can be leveraged to prevent delayed or overnight hypoglycemia, assist during prolonged activity, or enhance mild hypoglycemia treatment (22, 31, 34). Meanwhile, protein co-ingestion with carbohydrate amplifies postprandial glucose rises and has important implications for meal bolusing and exercise planning (24).

Together, these findings underline the importance of individualized approaches to protein intake in diabetes management. Future tools—such as an “aminoglucogenic index” that predicts the glycemic impact of various protein sources—may help individuals with both T1D and T2D better anticipate hormonal and glycemic responses, improving time-in-range, reducing hypoglycemic episodes, and supporting long-term metabolic stability (32).

### 7.2 Conclusion

Dietary protein stimulates the secretion of both insulin and glucagon; however, the response time and potency of each hormone is dependent on numerous factors. A higher dose of protein preferentially stimulates alpha cells, prompting a lower insulin-to-glucagon ratio and, in turn, elevated blood glucose (18). This phenomenon is more easily achieved when protein is consumed in a mixed meal. Conversely, when protein is consumed alone, a very high dose is typically required to produce a measurable blood glucose excursion (23, 24). The rate at which insulin and glucagon are released is further dependent on the protein’s absorption rate (32), which are themselves influenced by multiple factors as outlined in **Table 1**. Overall, the endocrine and glycemic response to protein ingestion is dependent on many factors, including but not limited to digestion rate, protein dose, macronutrient content, and individual physiology. By choosing protein sources with consideration of these elements, the glycemic response to protein can be modified to better suit the specific needs of the individual with T1D, T2D, or prediabetes.

### 7.3 Areas for further research

Further studies assessing the implications of the glycemic and endocrine response to dietary protein for diabetes management could focus on:

- Responses and implications specifically for prediabetes
- The effects of protein in advanced T2D while on insulin sensitizers such as metformin
- The impact of protein quality/completeness on acute glycemic and hormonal response
- Rates of rebound hypoglycemia after rescue snacks of varying macromolecule content
- Classifying absorption rates of common protein sources
- More research into pre-exercise protein intake on preventing hypoglycemia
- Research to establish standardized protein ‘doses’ for specific clinical scenarios in T1D, such as pre-bed snacks or pre-exercise supplementations
- Long-term randomized controlled trials examining the impact of replacing fast-digesting, BCAA-rich proteins with slow digesting proteins on insulin sensitivity in T2D
- Studies exploring the factors that predict interindividual variability in glycemic responses to different protein types
- More research into optimal AID system algorithms to account for meal composition
